# Establishment and Evaluation of a 6-Gene Survival Risk Assessment Model Related to Lung Adenocarcinoma Microenvironment

**DOI:** 10.1155/2020/6472153

**Published:** 2020-04-03

**Authors:** Zhitian Wang, Huiling Xu, Linhai Zhu, Tianyu He, Wang Lv, Zhigang Wu

**Affiliations:** Department of Thoracic Surgery, The First Affiliated Hospital, School of Medicine, Zhejiang University, China

## Abstract

**Objective:**

A survival risk assessment model associated with a lung adenocarcinoma (LUAD) microenvironment was established and evaluated to identify effective independent prognostic factors for LUAD.

**Methods:**

The public data were downloaded from the TCGA database, and ESTIMATE prediction software was used to score immune cells and stromal cells for tumor purity prediction. The samples were divided into the high-score group and the low-score group by the median value of the immune score (or stromal score). The Wilcoxon test was used for differential analysis. GO and KEGG enrichment analysis of differentially expressed genes (DEGs) was performed using “clusterProfiler” of R package. Meanwhile, univariate and multivariate regression analysis was performed on DEGs to construct a multivariate Cox risk regression model with variable gene expression levels as independent prognostic factors affecting a tumor microenvironment (TME) and tumor immunity.

**Results:**

This study found that LUAD patients with high immune cell (stromal cell) infiltration had better prognosis and were in earlier staging. Functional enrichment analysis revealed that most DEGs were related to the proliferation and activation of immune cells or stromal cells. A survival prediction model composed of 6 TME-related genes (CLEC17A, TAGAP, ABCC8, BCAN, FLT3, and CCR2) was established, and finally, the 6 feature genes closely related to the prognosis of LUAD were proved. The AUC value of the ROC curve in this model was 0.7, indicating that the model was reliable.

**Conclusion:**

Six genes related to the LUAD microenvironment have a predictive prognostic value in LUAD.

## 1. Introduction

According to CA statistics in 2018, lung cancer is the most common cancer worldwide (11.6% of total cases) and the leading cause of cancer deaths (18.4% of total cancer deaths) [1], which seriously threatens human health. Non-small-cell lung cancer (NSCLC) accounts for about 80% of lung cancer, which is further divided into three histological subtypes, including lung adenocarcinoma (LUAD), squamous cell carcinoma (LUSC), and large cell carcinoma (LCLC). LUAD is the dominant subtype of NSCLC, with a low overall survival (OS) [2]. In recent years, tumor immunotherapy has been a research hotspot in tumor therapy, and its efficacy is closely related to a tumor microenvironment (TME). Therefore, finding diagnostic, therapeutic, and prognostic targets related to the TME is critical to the implementation of precision medicine.

The TME includes various cell types (endothelial cells, fibroblasts, immune cells, etc.) and extracellular components (cytokines, growth factors, hormones, extracellular matrix, etc.) that are surrounding tumor cells and nourished by a vascular network [3]. Immune cells (macrophages, mast cells, neutrophils, etc.) and adaptive immune cells (T and B lymphocytes) in the TME interact with tumor cells by direct contact or through chemokine and cytokine signal transduction, which influence tumor behavior and response to treatment. Many scholars have found that immune cells can both improve and obstruct therapeutic efficacy and may vary in their activation status and localization within the TME [3]. Tumor-infiltrating lymphocytes (TILs), for example, inhibit tumor growth by directly killing the tumor cells, while preserving the immune escape of malignant cells in the tumor to promote tumor development [4, 5]. M1, which suppresses tumor development, and M2, which promotes tumor development, are the two subtypes of tumor-associated macrophages (TAMs) [6–8]. Stromal cells are another type of important cells in the TME, and numerous studies have indicated that stromal cells and tumor cells have a bidirectional, dynamic, and complex relationship [9]. For example, carcinoma-associated fibroblasts (CAFs) not only block antitumor drugs but also induce tumor resistance, which is closely related to poor prognosis of tumors [10]. The immune and stromal cells in the TME play important roles in the development of tumor. Mining-related genes and independent prognostic factors followed with studying their impacts on tumor development and prognosis will help to improve the cure rate of patients.

Our team finally screened 6 genes (CLEC17A, TAGAP, ABCC8, BCAN, FLT3, and CCR2) related to immune and stromal cells in the TME through bioinformatics and constructed a risk assessment model to predict the prognosis of LUAD and verified evaluation efficiency of the model, which provides new ideas for improving the prognosis of LUAD.

## 2. Methods and Materials

### 2.1. Public Data Were Downloaded from the TCGA Database

FPKM dataset of TCGA-LUAD mRNA was downloaded from the TCGA database (https://www.cancer.gov/about-nci/organization/ccg/research/structural-genomics/tcga), which contains 535 LUAD samples and 59 paracancerous samples.

### 2.2. ESTIMATE Score Method

The immune and stromal cells of each sample were scored by ESTIMATE software to predict the tumor purity, with method reference to the research [11]. In brief, we devised two gene signatures, a “stromal signature” and an “immune signature,” which were used to generate scores that reflected the presence of each cell type in tumor samples and to measure tumor purity. The samples were divided into the high-score group and the low-score group by the median value of the immune score (or stromal score). The Wilcoxon test was used for differential analysis on the groups, and the results were obtained by intersection.

### 2.3. Functional Enrichment and COX Analysis

The R package “clusterProfiler” was used to conduct GO and KEGG enrichment analysis on the differentially expressed genes (DEGs). At the same time, univariate and multivariate regression analysis was conducted on DEGs, and a multivariate Cox risk regression model with variable gene expression values was constructed as an independent prognostic factor affecting TME and tumor immunity.

### 2.4. ROC Analysis and Survival Curve Plotting

A survival analysis was performed on the 6-gene survival model to explore its potential prognostic value. The survival time of the high- and low-risk groups which were divided according to the median risk scoring value was compared using the Kaplan-Meier method, and the differences between survival curves were tested and evaluated by log-rank. ROC curves were plotted for the 6-gene model to calculate the AUC value.

## 3. Results

### 3.1. Immune and Stromal Scores Are Associated with Different Clinical Stages and Prognosis of LUAD

**Figure 1:**
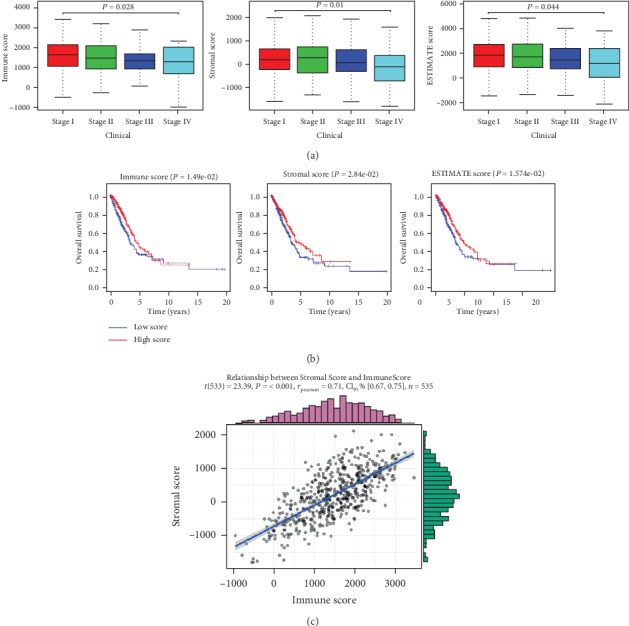
Immune and stromal scores were significantly correlated with different clinical stages and prognosis of LUAD. (a) The differences in different clinical stages of LUAD and (b) survival curves of the high-score (red line) and low-score (blue line) groups on the patients' prognosis of immune, stromal, and ESTIMATE scores were displayed. (c) Pearson's correlation coefficient of immune and stromal scores in tumor samples.

According to FPKM data of mRNA from TCGA-LUAD, the immune and stromal cells in each sample were scored by ESTIMATE, and the samples were divided into the high-score and low-score groups by a median score. It was found that the scores of immune cells in tumor samples significantly varied in different clinical stages of LUAD and were decreased with the increasing of stage according to the clinical information of TCGA-LUAD (*P* < 0.05). However, the scores of stromal cells were not related to the stage (*P* > 0.05) (Figure 1(a)). The infiltration degree of immune and stromal cells had a significant effect on the prognosis through survival analysis, which presented that the survival time of high infiltration patients was significantly longer than that of low infiltration patients (*P* < 0.05). (Figure 1(b)). Then, the results of Pearson's correlation coefficient analysis indicated that there was a remarkable positive correlation between immune and stromal scores in the samples (*P* < 0.05) (Figure 1(c)). The above results demonstrated that immune and stromal scores were significantly related to different clinical stages and prognosis of LUAD, and low-score patients were often accompanied by poor prognosis.

### 3.2. Mining DEGs of High Immune Score (Stromal Score) and Low-Score Groups

**Figure 2:**
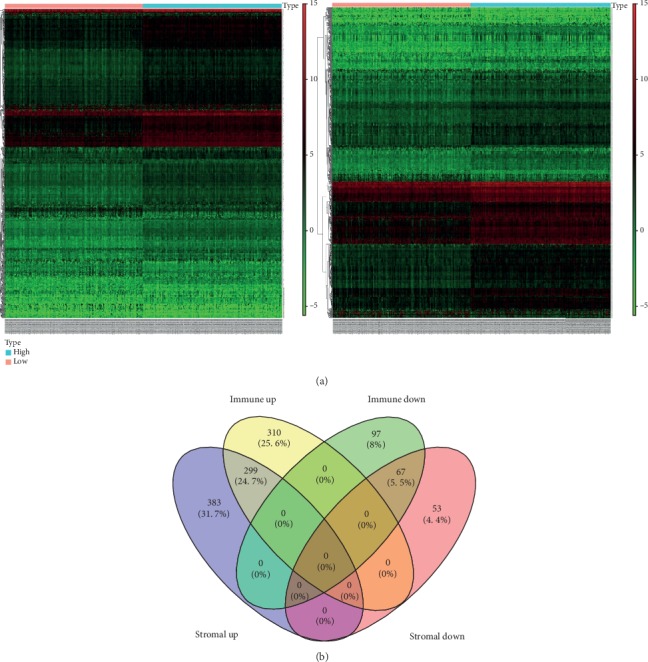
DEGs of the high immune score (stromal score) and low score groups were mined. (a) Heat maps of DEGs in the immune score (left) and stromal score (right) groups and (b) Venn diagram of DEGs in two groups.

The samples were divided into the high-score group and the low-score group by the median value of the immune score (or stromal score) and analyzed by the Wilcoxon test. The high immune score group obtained 611 upregulated genes and 164 downregulated genes, and the high stromal score group obtained 682 upregulated genes and 120 downregulated genes (Figure 2(a)). A total of 299 upregulated genes and 67 downregulated genes were obtained by the interaction of DEGs in two groups (Figure 2(b)).

### 3.3. Enrichment Analysis of DEGs

**Figure 3:**
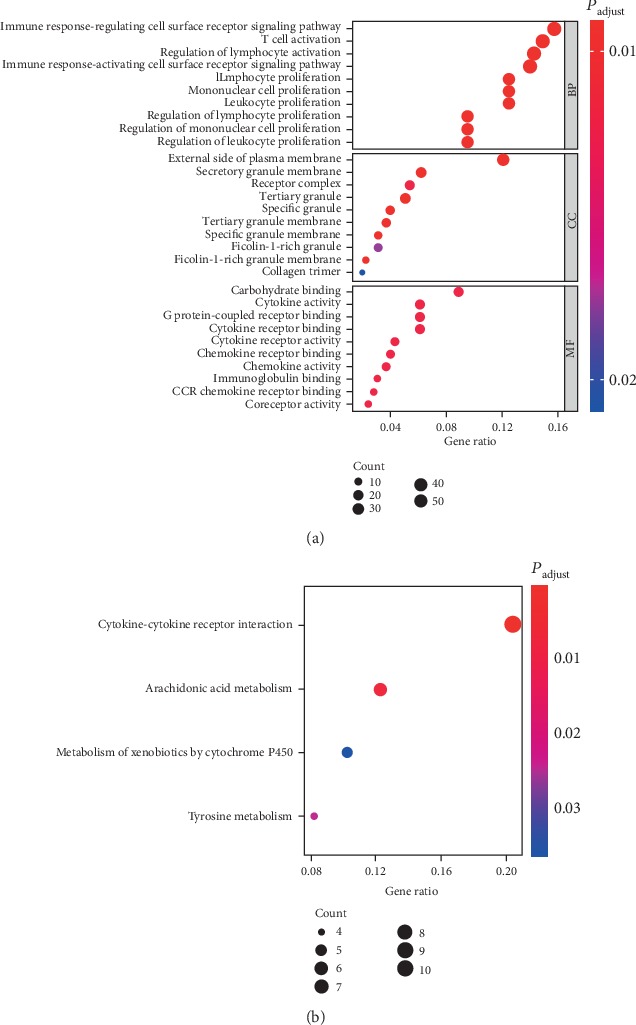
Enrichment analysis was performed on DEGs. (a) GO and (b) KEGG enrichment analysis results of DEGs at the intersection of two groups.

In order to further understand the functions of these DEGs in tumorigenesis and development, we used the R package “clusterProfiler” to perform GO and KEGG enrichment analysis on the DEGs (Figures 3(a) and 3(b)) and found that most genes were related to the proliferation and activation of immune or stromal cells, while immune and stromal cells were part of the TME. These TME-related genes were likely to affect tumor development by regulating the proliferation of microenvironmental cells.

### 3.4. Screening of Differential Genes with Independent Prognostic Value

**Figure 4:**
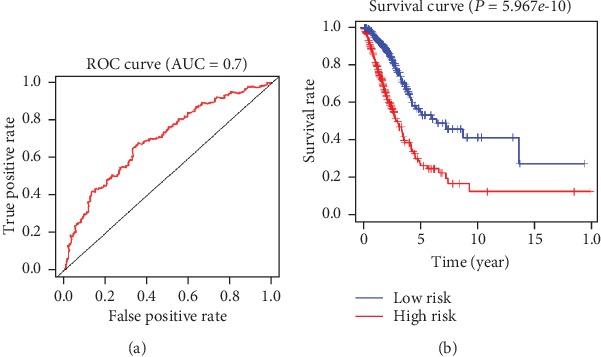
The reliability of the model constructed by DEGs with independent prognostic factor values was validated. (a) The ROC curves of the risk model, where the vertical axis represents the true-positive rate and the horizontal axis represents the false-positive rate. (b) The survival curves of the high- and low-risk groups in the risk model, where the red line represents the high-risk group and the blue line represents the low-risk group.

Univariate and multivariate Cox risk regression analysis was performed on 366 DEGs, and a multivariate Cox risk regression model was constructed based on survival time and survival status of patients. Six prognostic risk genes (CLEC17A, TAGAP, ABCC8, BCAN, FLT3, and CCR2) were finally screened as independent prognostic factors of LUAD. The risk assessment score formula was as follows: risk score = (‐0.29311)∗CLEC17A + (0.258641)∗TAGAP + (‐0.12533)∗ABCC8 + (0.169511)∗BCAN + (0.207814)∗FLT3 + (‐0.30955)∗CCR2. Then, we verified the model reliability through the ROC curves, and the results exhibited that the AUC value was 0.7, indicating that the model had certain accuracy (Figure 4(a)). Samples were split into the high-risk group and the low-risk group. The results of survival analysis revealed that the survival time of patients in the high-risk group was significantly shorter than that in the low-risk group (Figure 4(b)). The above results indicated that the TME-related 6-gene risk assessment model had predictive value for the prognosis of LUAD.

### 3.5. The Expression Level of Each Gene in the Risk Model Is Related to the Prognosis of Patients

**Figure 5:**
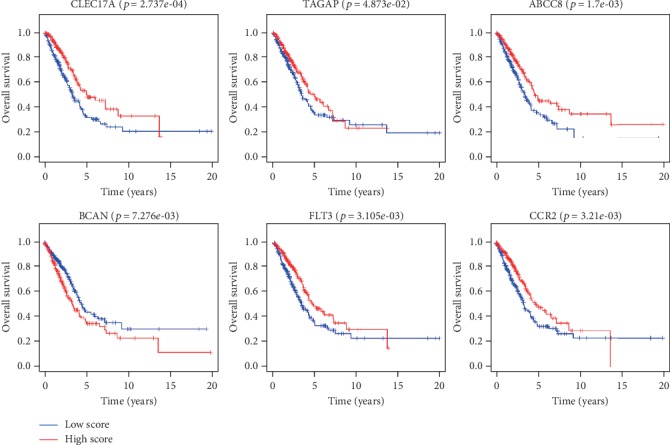
Expression levels of 6 genes in the risk model were related to the prognosis of patients. Survival curves of the 6 genes (CLEC17A, TAGAP, ABCC8, BCAN, FLT3, and CCR2) expressions in the high-expression (red line) and low-expression (blue line) groups in the risk model on the prognosis of patients.

In order to verify the correlation between each gene in the risk model and the prognosis, we divided the 6 genes into the high-expression group and the low-expression group according to the median value of their expressions and carried out survival analysis on these genes. The results displayed that the 6 genes had a significant impact on the prognosis of patients (Figure 5). Patients with high expressions of CLEC17A, TAGAP, ABCC8, FLT3, and CCR2 had a better prognosis and higher OS within 5 years than those with low expressions, while patients with high expression of BCAN had poorer prognosis and lower OS within 5 years.

## 4. Discussion

In this study, the immune and stromal cells in each sample were scored by ESTIMATE. The risk score was remarkably related to the tumor purity of clinical cancer and cancer cell line samples, and we provided an available and direct method to measure the number of tumor cells in biological samples. Tumor purity is the percentage of tumor cells in the TME and is significantly correlated with the clinical manifestations and prognosis of patients according to the researches in recent years. Zhang et al. [12] have studied the correlation between tumor purity and the prognosis of glioma and have reported that glioma purity is highly correlated with clinical and molecular features. Low purity cases are more likely to be diagnosed as malignant tumors and correlated with reduced survival time. The predictive validity can be significantly improved by integrating glioma purity into prognostic nomogram. Mao et al. [13] have discovered that tumor purity exhibits a potential value for colorectal cancer prognostic stratification as well as adjuvant chemotherapy benefit prediction. The relatively worse survival in low purity colorectal cancer may attribute to higher mutation frequency in key pathways and purity-related microenvironmental changing. Tumor purity also has a significant effect on tumor transcriptome. Rhee et al. [14] have obtained the expression profiles and tumor purity of 7,794 tumor specimen across 21 tumor types from the TCGA database and have found that immune genes are significantly inversely correlated with tumor purity. The expression of genes implicated in immunotherapy and specific immune cell genes, along with the abundance of immune cell infiltrates, is substantially inversely correlated with tumor purity. Tumor samples with lower tumor purity have more immune cells and tend to have a higher mutational load because the inflammatory response caused by immune cells can increase the mutation rate of tumor cells, and the effect of immunotherapy may be better [15]. In another study, researchers have used the GEO database of NSCLC cohort and ESTIMATE algorithm to estimate the immune score of tumor stromal cells and immune cells. Ultimately, they screened 10 genes out of 448 DEGs that were constructed as the risk prediction model. The ten-gene model was more sensitive to prognosis than TNM staging [16]. However, few researches have been carried out on the correlation between tumor purity and development of LUAD. In this study, we found that LUAD patients with high immune score (stromal score) had a better prognosis and higher OS than those with low score, while LUAD patients with high tumor purity were often accompanied by poor prognosis. We also studied the correlation between the ESTIMATE score and the LUAD staging and observed that the later the staging, the lower the score, suggesting that patients with low score tended to have more advanced and dangerous tumor.

The pathway enrichment analysis was performed on DEGs, and it was found that most genes were related to the proliferation and activation of immune or stromal cells. Immune cells interact with tumor cells through direct contact or signal transduction of chemokines and cytokines and influence tumor behavior and treatment response. Therefore, mining prognostic risk factors related to the TME is conducive to further improvement of immunotherapy. In order to further explore the genes related to prognosis, we obtained 6 TME-related genes through differential, regression, and enrichment analyses, namely, CLEC17A, TAGAP, ABCC8, BCAN, FLT3, and CCR2. A prediction model of these 6 genes was constructed, and the results showed that the OS of patients with a high risk score was significantly lower than that with low risk. The ROC curves were used to verify the model accuracy, and the AUC value was 0.7, indicating the model had certain accuracy. Gene enrichment analysis exhibited that these genes were related to the proliferation and activation of immune or stromal cells. Related literature has reported that these genes play an important role in the development of multiple diseases. For example, CLEC17A is a member of the calcium-dependent family (type C lectin), and the protein encoded by CLEC17A gene is mainly expressed in B cells dividing in the germinal center of secondary lymphoid organs, which is related to cell adhesion [17]. Single-nucleotide polymorphisms in TAGAP are associated with a variety of autoimmune diseases. Some researchers have put forward that TAGAP variation modulates the risk of autoimmunity by altering thymocyte migration during thymic selection [18]. Mutations in the ABCC8 gene are closely related to diabetes [19, 20]. BCAN-NTRK1 is an effective glioma driver and therapeutic target. Studies have simulated four relatively rare chromosomal rearrangements with unknown oncogenic potential in human brain glioma and have found that one of the chromosomal deletions results in fusion between BCAN and NTRK1, which promotes the formation of highly invasive glioma [21]. FLT3 mutations are associated with polyunsaturated fatty acid metabolism, and they play a previously underappreciated role in obesity-related leukemia [22]. CCL2 secreted from cancer-associated mesothelial cells promotes peritoneal metastasis of ovarian cancer cells through the P38-MAPK pathway [23]. CCL2 is highly expressed in M2 macrophages but antagonized by miR-511-3p, and miR-511-3p regulates allergic inflammation and macrophage polarization by targeting CCL2 and its downstream Ccr2/RhoA axis [24]. However, the role of these genes in LUAD development has not been reported. In this study, the expressions of these 6 genes were further examined by survival analysis, and the results suggested that these genes were significantly correlated with the prognosis. Patients with high expressions of CLEC17A, TAGAP, ABCC8, FLT3, and CCR2 had better prognosis and higher OS within 5 years, while patients with high expression of BCAN had poorer prognosis and lower 5-year OS.

In conclusion, we demonstrated through a series of rigorous analyses that tumor purity was closely related to LUAD development and that LUAD patients with high infiltration of immune cells (stromal cells) had better prognosis and earlier staging. Meanwhile, we identified 6 core genes closely related to the prognosis of LUAD and constructed a survival prediction model for TME-related genes. The AUC value of the ROC curves in this model was 0.7, which proved that the model was reliable. However, the study has not yet explored the correlation and molecular mechanism of the expressions of 6 genes and the development of LUAD, which still needs to be further investigated. These observations collectively provide a new idea for the prognosis of LUAD and a new direction for tumor immunotherapy.

## Data Availability

The data used to support the findings of this study are available from the corresponding author upon request.

## Additional Points

*Highlights*. (1) ESTIMATE score is used to evaluate tumor purity, and it is found that patients with high scores have better prognosis and are in earlier staging. (2) A 6-gene (CLEC17A, TAGAP, ABCC8, BCAN, FLT3, and CCR2) survival prediction model is constructed, and the AUC value of ROC curve is 0.7. (3) It is found that patients with high expressions of CLEC17A, TAGAP, ABCC8, FLT3, and CCR2 have a better prognosis, while patients with high expression of BCAN have a poorer prognosis.
